# Correction: The manifold structure of limb coordination in walking *Drosophila*

**DOI:** 10.7554/eLife.65214

**Published:** 2020-12-04

**Authors:** Brian D DeAngelis, Jacob A Zavatone-Veth, Damon A Clark

DeAngelis BD, Zavatone-Veth JA, Clark DA. 2020. The manifold structure of limb coordination in walking *Drosophila*. *eLife* **8**:e59795. doi: 10.7554/eLife.46409.

Published 28, June 2019

After publication, we became aware of an inadvertent sign error in the coherence analysis reported in the subsection “Template-matching suggests an absence of canonical tetrapod and wave gaits” of the Results and described in the subsection “Coherence analyses” of the Methods. The incorrect equation, listed as Equation (5) of the Methods and used in our analysis code, read

$$r\left(t\right)exp\left\{i\Phi \left(t\right)\right\}=\frac{1}{6}\sum_{k=1}^{6}exp\left\{i\left(\phi_{k}\left(t\right)+\psi_{k}\right)\right\};$$

The corrected equation reads

$$r\left(t\right)exp\left\{i\Phi \left(t\right)\right\}=\frac{1}{6}\sum_{k=1}^{6}exp\left\{i\left(\phi_{k}\left(t\right)-\psi_{k}\right)\right\}.$$

This sign error did not alter any tripod coherence values, but correcting this error caused the tetrapod and wave coherences to become larger on average relative to the incorrect analysis. This affected some qualitative descriptions of the data, but not the conclusions we drew from these analyses. We thank Adam Gosztolai for alerting us to this error, and we regret any confusion resulting from it. We have corrected the manuscript through the following changes:

First, the equation in the Methods has been corrected, as has the equation in the analysis code online.

Second, the manuscript text has been revised to reflect the corrected analyses. We show below the original and corrected versions of the two pertinent sections of text.

Original section of text from the Results:

## Template-matching suggests an absence of canonical tetrapod and wave gaits

We next sought to systematically investigate how closely our data matched the configurations of relative limb phasing specified by each of the canonical gaits. To do so, we defined a template-matching coherence metric based on a simple model for networks of coupled oscillators (see Materials and methods). The coherence falls between zero and one, with unity corresponding to an exact match to the template phase configuration. The tripod coherence distribution is sharply peaked around ~0.9, indicating a close template match, while the distributions for the other canonical gaits were broader and peaked at lower coherences (Figure 3H). If the fly preferentially used different gaits at different forward walking speeds, then we would expect to see a peak in the conditional coherence distribution for a given canonical gait at some characteristic forward velocity. Yet, we did not observe any such distinct peaks in our data (Figure 3I). At all but the lowest speeds, the most coherent phase template is tripod (Figure 3J). Furthermore, the coherences in non-tripod modes are quite low, even among those instances better described by canonical gaits other than tripod (Figure 3—figure supplement 3). Therefore, under an analysis designed to sensitively compare our data to canonical gait configurations, we do not observe significant evidence for the use of canonical tetrapod and wave gaits.

Corrected section of text:

## Template-matching suggests little evidence for preferred canonical tetrapod and wave gaits

We next sought to systematically investigate how closely our data matched the configurations of relative limb phasing specified by each of the canonical gaits. To do so, we defined a template-matching coherence metric based on a simple model for networks of coupled oscillators (see Materials and methods). The coherence falls between zero and one, with unity corresponding to an exact match to the template phase configuration. The tripod coherence distribution is sharply peaked around ~0.9, indicating a close template match, while the distributions for the other canonical gaits were broader and peaked at lower coherences (Figure 3H). If the fly preferentially used different gaits at different forward walking speeds, then we would expect to see a peak in the conditional coherence distribution for a given canonical gait at some characteristic forward velocity. Yet, there were not prominent peaks in our data, and all coherences yielded low average values at slow walking speeds (Figure 3I). At those slower speeds, where mean coherences were low, the tetrapod coherences became more likely to be the highest coherence (Figure 3J). This is consistent with the observation of tetrapod-like coordination patterns at low speeds (Figure 3F–﻿G), and with the increased spread of the distribution of relative phases at low speeds (Figure 3D,E, Figure 3—﻿figure supplements 2 and 3; Wosnitza et al., 2013). However, even when the largest coherence was not tripod, these non-tripod coherences were lower than when the tripod coherence was largest (Figure 3—figure supplement 3). Since the tetrapod coherence of a perfect tripod gait is 3^–1/2^ ~ 0.6, this coherence metric may not show strong contrasts under some deformations in coordination. Therefore, under these analyses, we do not observe strong evidence for the preferred use of canonical tetrapod and wave gaits.

The corrected version of Figure 3 is shown here:

**Figure 1:**
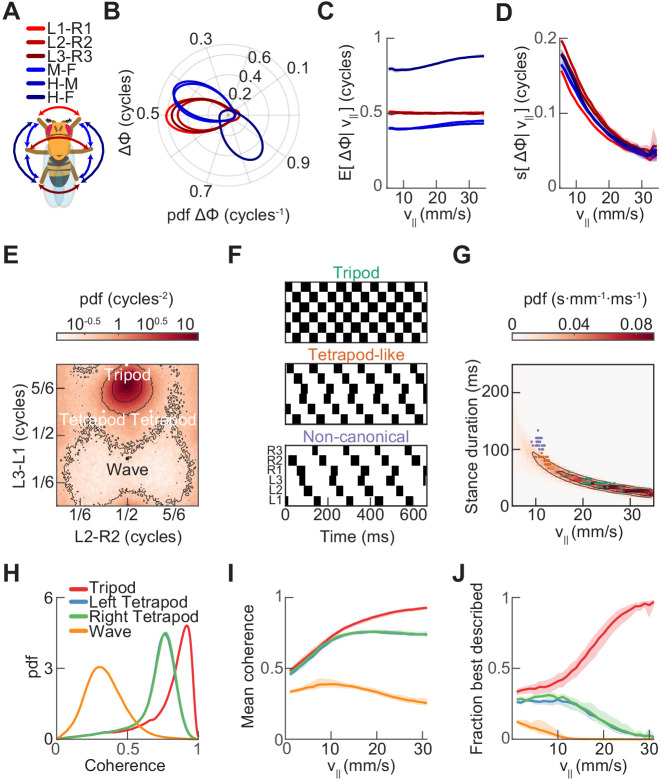


The originally published version of Figure 3 is shown for reference:

**Figure 2:**
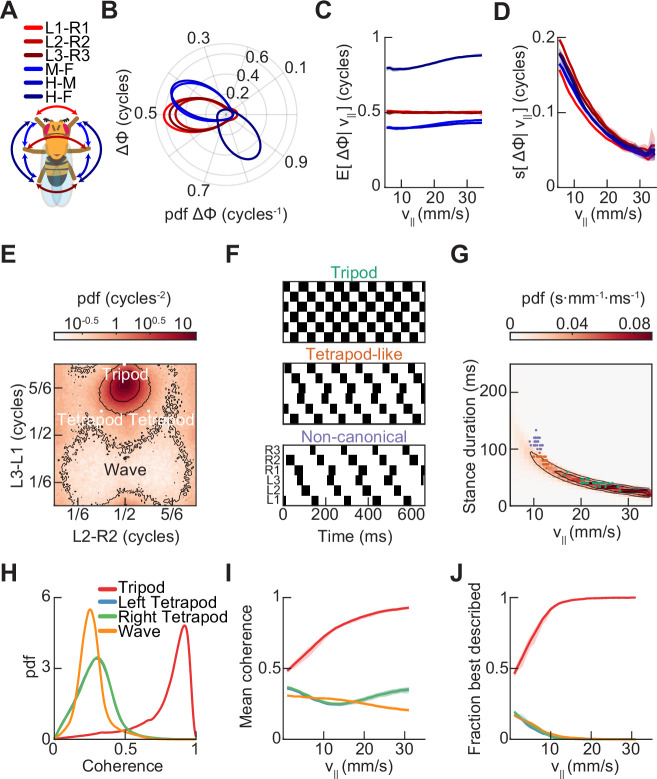


The corrected version of Figure 3 – figure supplement 3 is shown here:

**Figure 3:**
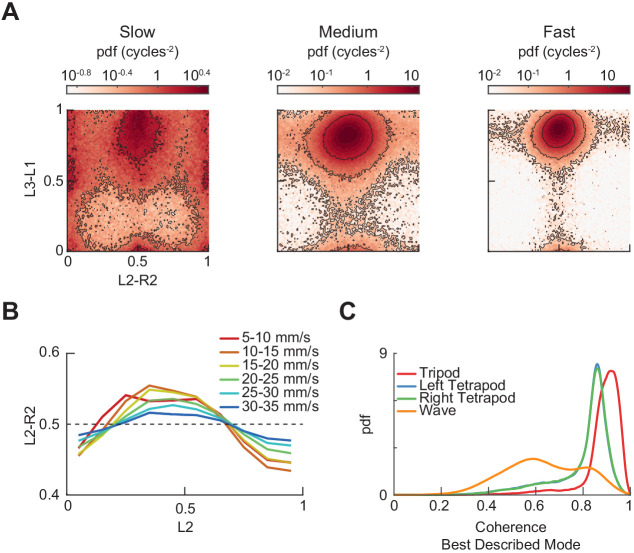


The originally published version of Figure 3 – figure supplement 3 is shown for reference:

**Figure 4:**
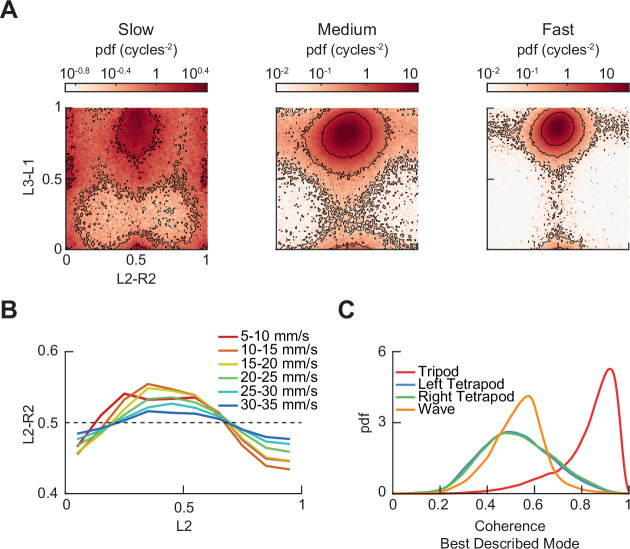


The article has been corrected accordingly.

